# The “Cone” stoma: stoma creation in the presence of significant bowel dilatation

**DOI:** 10.1007/s10151-018-1924-9

**Published:** 2019-03-01

**Authors:** A. lo Conte, K. Wasmann, P. J. Tanis, C. J. Buskens, R. Hompes, W. A. Bemelman

**Affiliations:** Department of Surgery, Amsterdam UMC, University of Amsterdam, Meibergdreef 9, 1105 AZ Amsterdam, The Netherlands

## Introduction

**Fig. 1:**
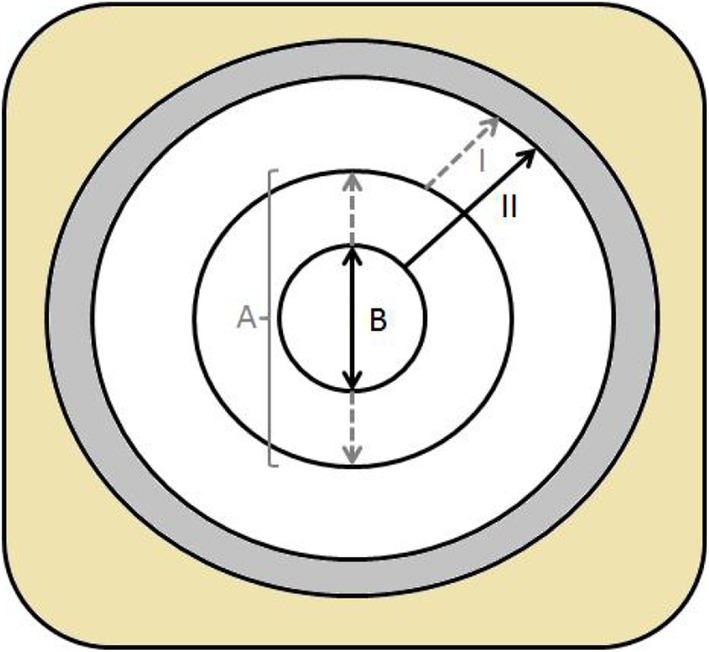
Ostomy plate. Large stoma: A represents the pouch stoma opening, I: minimal residual adhesive border. “Cone stoma”: B is the reduced pouch stoma opening, II: more adhesive margin

The creation of either an ileostomy or colostomy is usually perceived as a quick routine “act”; however, considering the potential impact on the patient’s outcome and quality of life, it deserves the utmost attention to detail, particularly if the stoma will be permanent [1]. Stoma-related complications range from 20 to 70%, and span a great variety of problems [2]. Parastomal hernias occur in up 50% of patients with variable incidence according to stoma type and configuration; 1.8–28.3% and 0–6.2% for end and loop ileostomies, and 4–48% and 0–30.8% for end and loop colostomies, respectively [3, 4]. Stoma prolapse, defined as a full-thickness protrusion of bowel through the stoma, occurs in 2–11% of ileostomies and 2% of colostomies [2]. These are usually both considered late complications, and are associated with older age, obesity, surgical technique, and bowel obstruction. Stoma ischemia/necrosis, retraction, mucocutaneous separation, and parastomal abscess are considered the early complications, and good surgical technique plays a critical role in preventing them. The formation of a “good” stoma is more complex in case of acute bowel obstruction or chronically distended bowel. The dilated bowel requires a large opening in the abdominal wall and results in a wide skin defect and a large mucocutaneous circumference. For any pouching system to fit, a large central opening needs to be cut which reduces the surface for skin adhesion of the pouch baseplate, which inevitably will cause the leaks of stoma content and skin irritation (Fig. 1).

Here, we describe our technique to create a normally sized stoma in patients with a significant bowel dilatation.

## Surgical technique

An elliptical surgical incision is performed at the previously marked site in the right or left lower quadrants of the abdomen according to the type of stoma.

After subcutaneous dissection, the anterior fascia is incised and the rectus abdominis muscle is separated parallel to its fibers to expose the posterior sheath while avoiding the epigastric vessels. The posterior sheath is carefully incised to avoid injury to underlying bowel. The aperture in the abdominal wall should be generally sized to accommodate the stoma without engorgement of the mesentery.

**Fig. 2:**
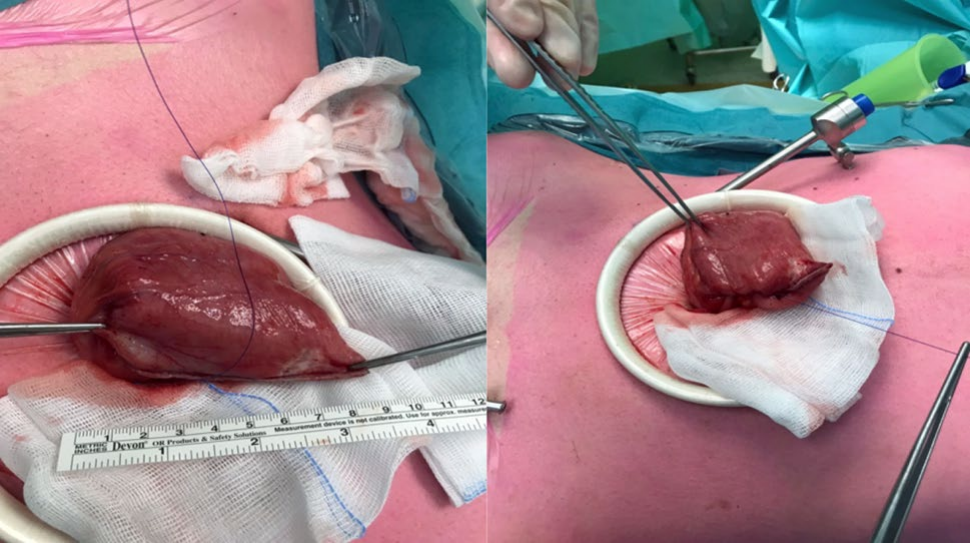
On the right side: the dilated bowel is extracted from the abdomen. On the left: the stapler line is over-sewn leaving 2–3 cm unaffected

The dilated bowel (Fig. 2) previously transected with a stapler is extracted through an Alexis wound retractor (Applied Medical, Rancho Santo Margarita, CA, USA).

The stapler line is over-sewn using a polydioxanone (PDS, Ethicon) suture, leaving 2–3 cm unaffected (Fig. 2).

**Fig. 3:**
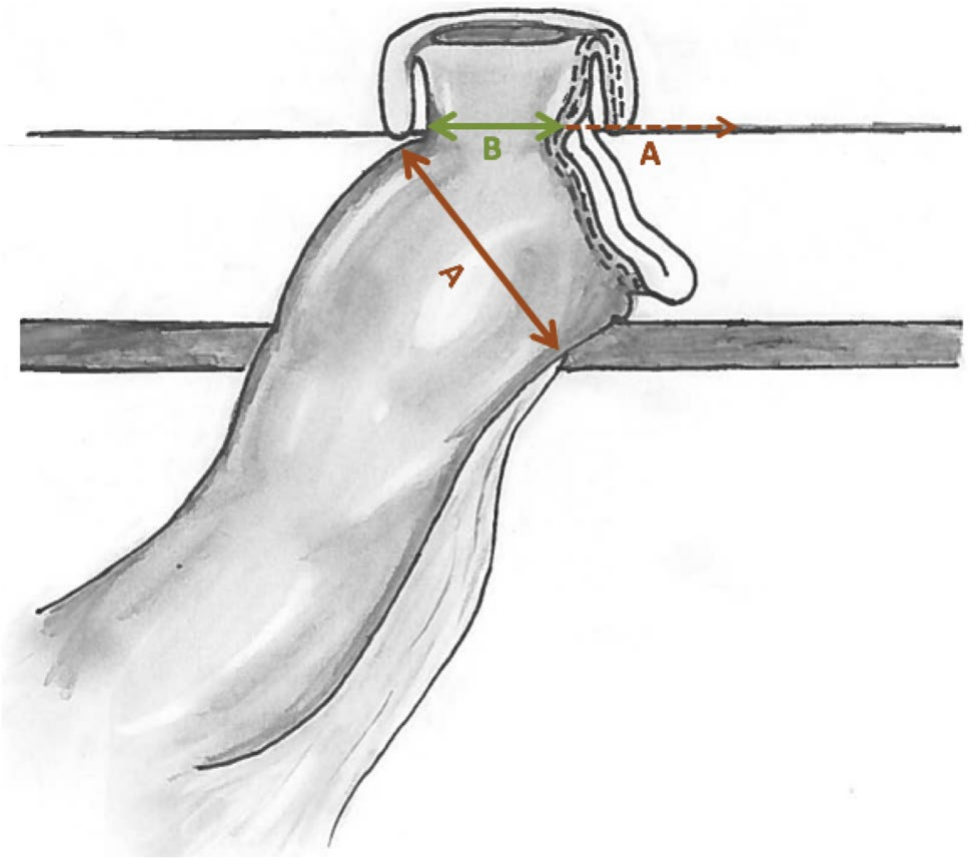
Continuous line A indicates the diameter of the dilated bowel. Continuous line B is the reduced length of the not over-sewn segment, the only to be exteriorized and used to performed the “Cone” stoma. Dotted line A is the length of the over-sewn bowel segment. Line B + dotted line A represents the eventually stoma size with the classical technique

**Fig. 4:**
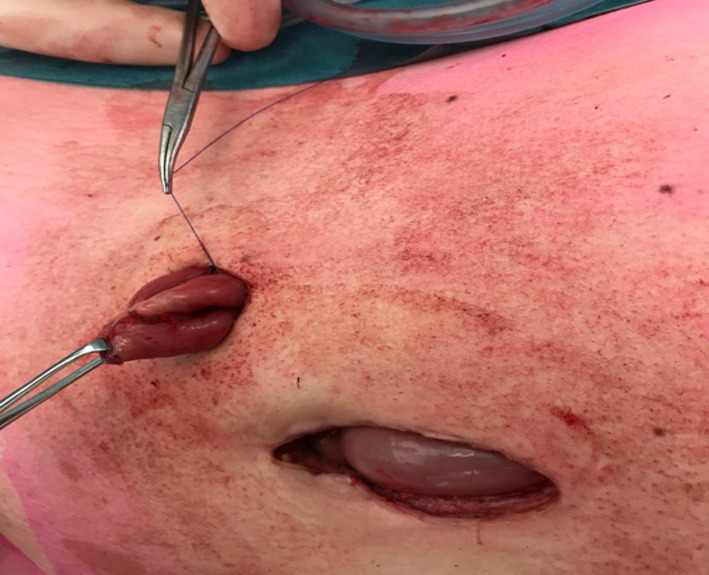
The not over-sewn segment is the only one to be exteriorized (continuous line B in the drawing)

**Fig. 5:**
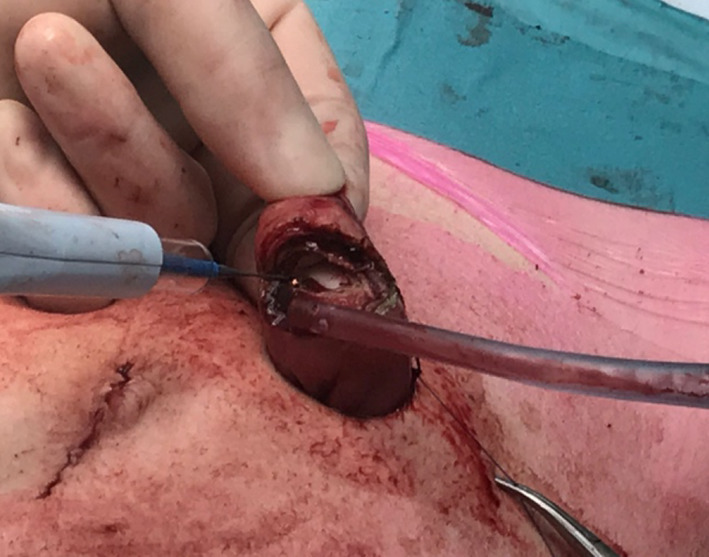
Incision of the external tip

The tip of the stapler line (the not over-sewn segment, B) (Figs. 3, 4) is the only segment finally exteriorized through the skin opening once the wound retractor is released. Using electrocautery, the bowel is cut at the exteriorized tip (Fig. 5).

**Fig. 6:**
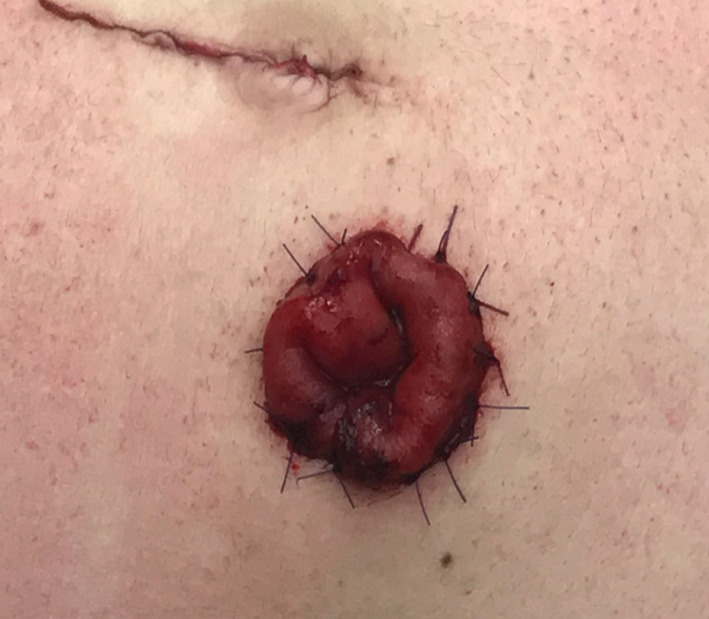
Stoma is fixed to the skin with PDS 3/0 stitches

Full-thickness eversion of the bowel wall is then performed to create a 2 cm spout and fixation of the stoma to the skin with PDS3/0 stitches. As such, a cone stoma is created with a normal diameter avoiding a very wide and potentially troublesome stoma (Fig. 6).

## Conclusions

This technique is proposed to construct a stoma in cases of chronic bowel obstruction avoiding a large and redundant stoma.

It is a simple and feasible procedure, with better aesthetic and functional results than the classical technique as well as resulting in better patient acceptance.
